# Metabolic Signature in Combination with Fecal Immunochemical Test as a Non-Invasive Tool for Advanced Colorectal Neoplasia Diagnosis

**DOI:** 10.3390/cancers17142339

**Published:** 2025-07-15

**Authors:** Oihane E. Albóniga, Joaquín Cubiella, Luis Bujanda, Patricia Aspichueta, María Encarnación Blanco, Borja Lanza, Cristina Alonso, Juan Manuel Falcón-Pérez

**Affiliations:** 1 Metabolomics Platform, CICbioGUNE-BRTA, Centro de Investigación Biomédica en Red de Enfermedades Hepáticas y Digestivas (CIBERehd), Bizkaia Technology Park, 48160 Derio, Spain; 2 Grupo de Investigación de Oncología Digestiva-Ourense, Department of Gastroenterology, Hospital Universitario de Ourense, Centro de Investigación Biomédica en Red de Enfermedades Hepáticas y Digestivas (CI-BERehd), Complexo Hospitalario Universidad de Ourense, 32005 Ourense, Spain; joaquin.cubiella.fernandez@sergas.es; 3 Department of Gastroenterology, Donosti Hospital, Universidad del País Vasco (UPV/EHU), 20014 San Sebastián, Spain; luis.bujandafernandezdepierola@osakidetza.eus; 4 Centro de Investigación Biomédica en Red de Enfermedades Hepáticas y Digestivas (CIBERehd), Biodonostia Health Research Institute, 20014 San Sebastián, Spain; patricia.aspichueta@ehu.eus; 5 Department of Physiology, Faculty of Medicine and Nursing, University of the Basque Country UPV/EHU, 48940 Leioa, Spain; 6 Biobizkaia Health Research Institute, 48903 Barakaldo, Spain; 7 OWL Metabolomics (Rubió Metabolomics S.L.U.), Bizkaia Technology Park, 48160 Derio, Spain; eblanco@owlmetabolomics.com (M.E.B.); blanza@owlmetabolomics.com (B.L.); calonso@owlmetabolomics.com (C.A.); 8 Exosomes Laboratory, CIC bioGUNE-BRTA, CIBERehd Centro de Investigación Biomédica en Red de Enfermedades Hepáticas y Digestivas (CIBERehd), Bizkaia Technology Park, 48160 Derio, Spain; 9 IKERBASQUE, Basque Foundation for Science, 48011 Bilbao, Spain

**Keywords:** cholesteryl esters, colorectal cancer, fecal extract, semi-targeted metabolomics

## Abstract

**Background/Objectives**: Colorectal cancer (CRC) is one of the most prevalent cancers worldwide. Even though the screening programs have decreased the incidence rates, the prognosis for CRC varies depending on the stage at diagnosis. Thus, early diagnosis is still a big challenge due to screening methods, and subsequent diagnosis is not very sensitive. **Methods**: In this work, LC-MS-based metabolomics, a powerful and sensitive tool to study complex dynamic changes, was used to analyze 211 human fecal samples from control individuals (CTRL), adenoma (AA), and CRC patients. **Results**: Multivariate and univariate statistical analysis highlighted cholesteryl esters (CEs) and fecal haemoglobin, quantified by fecal immunochemical test (FIT), as relevant biomarkers that clearly differentiate CRC from AA and CTRL. Predictive models based on random forest and the area under the curve (AUC) of the receiver operating characteristic curve (ROC) demonstrate that CEs, together with FIT measurement, improved the CRC and CTRL classification, but not AA. This study revealed that the AA group is a transitional stage with high heterogeneity. The increased tendency observed in CEs from CTRL to CRC might be related to the imbalance of cholesterol homeostasis due to cancer cells requiring a high cholesterol level for cell development and proliferation. The free cholesterol is probably obtained from CEs, as it is the most cost/effective way to obtain the needed cholesterol. **Conclusions**: The accumulation of CEs is produced by two possible approaches: (1) dysfunction of cholesterol absorption in the small intestine and/or (2) transported inside exosomes from cell to cell to promote proliferation.

## Simple Summary

Screening programs have decreased the incidence rates of colorectal cancer (CRC), but more efforts are needed for an early diagnosis. For this purpose, we perform a metabolomics analysis in fecal sample from three groups of patients: healthy individuals (CTRL), adenocarcinoma (AA) and CRC. In this study, groups CTRL and AA, as well as CTRL and CRC, are clearly separated and the results obtained allow us to enhance the accuracy of CRC diagnosis by adding cholesteryl esters (CEs) as biomarkers to the current diagnosis tool fecal immunochemical test (FIT). In addition, our results support the study of cancer metabolism using LC-MS-based metabolomics for the identification of sensitive and accurate biomarkers and potential therapeutic targets.

## 1. Introduction

According to the World Health Organization (WHO; 2024), colorectal cancer (CRC) is the third most common cancer worldwide, accounting for 9.6% of all cancer cases, and is the second leading cause of cancer-related deaths [1].

CRC is a type of cancer that affects and develops in the colon or rectum, in which abnormal cells divide uncontrollably, ultimately forming a malignant tumor [2]. The risk of CRC increases with age, being people over 50 years the most affected. The most common symptoms include diarrhoea, blood in the stool, abdominal pain, and low iron levels, among others. However, many people will not have symptoms in the early stages of the disease, and thus, regular screenings are crucial for early diagnosis [3].

Even though the incidence rates have decreased mainly due to effective screening programs, the prognosis for CRC varies depending on the stage at diagnosis. In this sense, early-stage cancers have higher survival rates than advanced-stage cancers [2,4]. Improving the early diagnosis rate of CRC is key to decreasing the case fatality and improving patients’ quality of life. The current screening tests are fecal occult blood test (FOBT), sigmoidoscopy, colonoscopy, virtual colonoscopy, and DNA stool test. Among them, FOBT is the most common screening tool. Two types of FOBT screening test are used, the fecal immunochemical test (FIT) that uses antibodies directed against human haemoglobin (Hb) in small stool samples and provide a quantitative value for fecal Hb, and guaiac-based fecal occult blood that looks for hidden blood in stool and reacts to guaiac substance (colorimetric method) [5,6]. Despite the advances, these methods are not very sensitive or accurate due to blood in stool means there is bleeding happening somewhere in the digestive tract, but it requires additional tests, such as a colonoscopy, to determine where the bleeding comes from [7]. For example, bleeding is usually a sign of a health condition such as diverticulosis, haemorrhoids, colon polyps, ulcers, as well as CRC.

Metabolomics, which is a powerful and sensitive tool to analyze complex dynamic metabolic changes, can help in the improvement of CRC early diagnosis. Several studies and reviews have been published in which metabolomics was used as an analytical approach to study and detect CRC biomarkers, providing a valuable body of knowledge for future diagnostic developments. Among the identified biomarkers, amino acids, fatty acids, lysophosphatidylcholines, cholesteryl esters, and acylcarnitines, among others, were found to be dysregulated in CRC patients. Most studies were mainly conducted using plasma, serum, or urine, with far fewer focusing on fecal samples [8,9,10].

Considering that one of the main advantages of metabolomics is its close reflection of the individual phenotype and its capacity of working with non-invasive samples, this technique offers significant potential for studying, identifying, and verifying CRC putative biomarkers previously found in both fecal and FIT extract samples, such as cholesteryl esters, sphingolipids or glycerophospholipids [11,12].

In the current study, endogenous lipidomic profiles of human fecal samples taken from healthy patients and patients with different degrees of colon pathologies (advanced adenoma (AA) and colorectal cancer (CRC)) have been studied using ultra-high performance liquid chromatography–mass spectrometry (UHPLC-MS). Furthermore, bearing in mind the lack of sensitivity of FIT [13], data of false positives and false negatives in FIT were also considered to study the accuracy of lipidomics profiles and/or lipids compared to the current diagnosis tools. Lastly, this study was used to determine possible phenotypical biomarkers by statistical analysis that could enhance the sensitivity for CRC early diagnosis, as well as compare them with previous findings.

## 2. Materials and Methods

### 2.1. Chemicals

HPLC-MS grade solvents were purchased from Sigma Aldrich (St. Louis, MO, USA) and Fisher Scientific (Pittsburgh, PA, USA). Reference metabolite standard compounds were obtained from Sigma Aldrich, Larodan Fine Chemicals (Malmö, Sweden) and Avanti Polar Lipids (Alabaster, AL, USA).

### 2.2. Clinical Samples and Study Population

A total of 211 human fecal samples were collected from the Hospital Universitario de Ourense (Galicia, Spain) and classified according to patient’s diagnosis based on colonoscopy as control (CTRL; N = 78), adenoma (AA; N = 58), or colorectal cancer (CRC; N = 75). The study was conducted according to the clinical and ethical principles approved by the Clinical Research Ethics Committee of Galicia (Code 2011/038). All participants provided written informed consent.

Samples were randomly selected from the public health repository data, where the inclusion criteria were asymptomatic individuals with intermediate risk. That is, individuals who do not have clinical symptoms, and patients who do not have any family history or other factors associated or related to colorectal cancer. Exclusion criteria were age under 18, pregnancy, asymptomatic individuals who were undergoing colonoscopy for CRC screening, patients with a previous history of colonic disease, patients requiring hospital admission, patients whose symptoms had ceased within 3 months before evaluation, and patients who declined to participate after reading the informed consent form.

Patients and control individuals self-collected a fecal sample from one bowel movement without specific diet or medication restrictions at home, the week before a colonoscopy was performed, and delivered it to the hospital. The fecal sample was sent to the laboratory in less than 4 h, split into aliquots, and immediately frozen at −80 °C. An additional sample was collected using the FIT collector and fecal haemoglobin concentration was assessed using the automated OCsensor™ (Eiken Chemical Co., Tokyo, Japan). Fecal haemoglobin concentration was considered positive if ≥20 µg Hb/g feces.

### 2.3. Metabolite Extraction

There is no single platform or method to analyze the entire metabolome in a biological sample due to the wide range of polarities, extensive chemical diversity, and concentrations among metabolites [14,15]. Therefore, the metabolite extraction used was based on the interest in profiling the lipid classes glycerolipids (GLs), sterol lipids (STs), sphingolipids (SPs), and glycerophospholipids (GPs). In this sense, the extraction was performed by using an appropriate combination of organic solvents and analyzing them in a specific UHPLC-MS platform optimized for extensive coverage of human samples’ lipidome. To extract those lipids of interest in fecal samples, an adapted protocol of serum samples was used [11,16].

Briefly, 15 mg of fecal extracts were mixed with 45 µL sodium chloride (50 mM) and spiked with 110 µL of chloroform/methanol (2:1) containing metabolites not detected in unspiked human normal extracts [SM (d18:1/16:0), PE (17:0/17:0), PC (19:0/19:0), TAG (13:0/13:0/13:0), Cer (d18:1/17:0), and CE (12:0)]. Samples were then vortexed and incubated for 1 h at −20 °C. After centrifugation at 18,000× *g* for 15 min at 4 °C, 50 µL of the organic phase was collected, and dried under vacuum. The dried extracts were then reconstituted in 100 µL of acetonitrile/isopropanol (1:1), centrifuged (18,000× *g* for 5 min), and transferred to vials for UHPLC-MS analysis. This extraction method allowed the optimal profiling of the previously mentioned lipid classes.

Additionally, different types of quality control (QC) samples were used to assess data quality; any drifts associated with the analysis were normalized and any time-related effects were corrected. The QC calibration sample was a reference serum sample used to correct the different response factors between and within batches. Also, a QC validation sample was prepared by pooling all fecal samples included in the study to assess how well the data pre-processing procedure previously performed with QC calibration improved the data quality. Both QC calibration and QC validation were extracted and analyzed at the same time as the individual samples. Two types of blanks, QC blank sample, prepared following the same extraction procedure as biological samples, and QC system suitability blank sample, prepared with the solvents used for biological samples reconstitution, were also included and analyzed.

For the analytical sequence, randomized sample injections were performed with each of the QC calibration and validation extracts uniformly interspersed throughout the entire batch run.

### 2.4. UHPLC-MS Metabolic Profiling

Metabolomics analysis of fecal extracts was performed by Rubió Metabolomics, S.L.U., previously OWL Metabolomics (Bizkaia, Spain), using an ACQUITY UPLC system coupled to a Xevo G2 QTOF mass spectrometer, both from Waters Corp. (Milford, MA, USA) and equipped with an electrospray ionization source operating in positive ionization mode (ESI+). Chromatographic separation was carried out by injecting 3 µL of sample on an ACQUITY UPLC BEH C18 column (2.1 × 100 mm, 1.7 µm), at 60 °C and a flow rate of 0.4 mL/min. A binary solvent system consisting of H_2_O/acetonitrile (2:3) with 10 mM ammonium formate (solvent A) and acetonitrile/IPA (1:3) with 10 mM ammonium formate (solvent B) was used for the elution. The gradient started from 60% A and 40% B, with a 10-minute linear gradient increasing from 40% to 100% B. After 5 min at 100% B, the mobile phase was reset to the initial composition in readiness for the subsequent injection to a total run time of 17 min. The mass spectra data were acquired in positive ionization mode with capillary and cone voltages of 3200 and 30 V, respectively. The desolvation gas was set to 1000 L/h at a temperature of 500 °C. The cone gas was set to 30 L/h, and the source temperature was set to 120 °C. The MS detector operated in centroid acquisition mode for a *m*/*z* range of 50–1200, using an accumulation time of 0.5 s per spectrum. All spectra were mass corrected in real time by reference to leucine enkephalin, infused at 10 µL/min through an independent reference electrospray, sampled every 10 s. The overall quality of the analysis procedure was monitored using repeat extracts of the QC samples. The retention time stability and mass accuracy were controlled and checked during analysis.

### 2.5. Data Pre-Processing

All data were processed using the TargetLynx application manager for MassLynx 4.1 software (Waters Corp., Milford, MA, USA). A set of predefined retention time–mass-to-charge ratio pairs, RT-*m*/*z*, corresponding to metabolites included in the analysis, was input into the program. Associated extracted ion chromatograms (EICs; mass tolerance window = 0.05 Da) were then peak-detected and noise-reduced in both the LC and MS domains such that only true metabolite-related features were processed by the software. A list of chromatographic peak areas was then generated for each sample injection.

For metabolites that were identified based on an in-house library built with pure standards and MS/MS analysis, representative MS detection response curves were generated using an internal standard for each chemical class included in the analysis. By assuming similar detector response levels for all metabolites belonging to a given chemical class represented by a single standard compound, a linear detection range was defined for each identified metabolite. Maximum values were defined as those at which the detector response became non-linear with respect to the concentration of the representative internal standard. Variables not considered for further analysis, where more than 30% of data points were found outside their corresponding linear detection range, were removed.

### 2.6. Data Normalization and Quality Control

Instrumental drifts in MS-driven metabolomics analysis have to be taken into consideration. In this sense, an intra- and inter-batch normalization based on multiple internal standards and a pool calibration sample approach was used. QC calibration samples were used for this purpose, following the procedure described previously [17]. Normalization factors were calculated for each metabolite by dividing its intensity in each sample by the intensity of an appropriate internal standard in that same sample. In this sense, the most appropriate internal standard for each variable was defined as that which resulted in a minimum relative standard deviation after correction, as calculated from the QC calibration samples over all the analysis batches. In this particular case, and following the pipeline established in Rubió Metabolomics, S.L.U., previously OWL Metabolomics (Bizkaia, Spain), the QC calibration was used as it was a well-controlled sample that could be used for future studies to compare data. Once the internal standard correction was carried out, a possible random or drift distribution in the QC calibration samples along the batch was assessed for each variable. For this, robust linear regression (internal standard corrected response as a function of sample injection order) was used to estimate in the QC calibration samples any intra-batch drift not corrected by internal standard correction. For all variables, internal standard corrected response in each batch was divided by its corresponding intra-batch drift trend, such that normalized abundance values of the study samples were expressed with respect to the batch averaged QC calibration serum sample (arbitrarily set to 1). Finally, the assessment of reproducibility was calculated using the QC validation samples of each batch to determine if normalization and corrections performed with the QC calibration could be extrapolated to QC validation samples and study samples [17].

### 2.7. Statistical Analysis

Once the data was normalized by each appropriate IS and the trend was corrected, statistical analysis was performed following different complementary approaches. First, age and gender were studied to determine group homogeneity using the PSPP tool (https://www.gnu.org/software/pspp/, accessed on 10 November 2024) by one-way ANOVA and post hoc tests, and then multivariate and univariate analyses were performed [18]. Also, FIT was treated as a categorical variable (yes or no) and as numerical, following the same approach to determine differences among groups.

Normality was also assessed by the Shapiro–Wilk test, applying a normality test to each metabolite to further perform univariate statistical analysis by a parametric Student’s t-test or a non-parametric Wilcoxon–Mann–Whitney test. To help visualization, different heatmaps were generated displaying the results of the univariate data analysis for each comparison. The heatmaps display the log2 (fold-change) of the metabolite included in the analysis, together with the *p*-value. For each metabolite, changes between subgroups were calculated as the base 2 logarithm of fold-change.

The multivariate data analysis was performed as a complementary tool to reduce the dimensionality of the complex data set to enable easy visualization of any clustering of the different groups of samples, as well as to detect outliers. This was achieved by principal component analysis (PCA). Model quality was evaluated by the explained variance (R2) and the predicted variance (Q2). R2 and Q2 had to be as high as possible and the cut-off value of the difference between both variances to consider a good PCA model should be that Q2 value was over 20% of R2 or the difference between them was less than 0.3 [19]. Different labels were used for tendency, such as main group (CRC, AA, or CTRL), gender, and FIT (yes or no). Then, supervised methods, such as partial least squares discriminant analysis (PLS-DA) and orthogonal PLS-DA (OPLS-DA), were used for classification and variable selection after an appropriate model validation criterion such as cross—validated analysis of variance (CV-ANOVA) [19]. In this sense, a variable importance on projection (VIP) score and an absolute value of *p*(corr) greater than 1.0 and 0.5, respectively, were used as cut-off points for variable selection [20].

All the statistics were performed using the Umetrics SIMCA-P software version 13.0.1 (Umetrics, Umea, Sweden) and RStudio (version 4.4.1). The predictive model analysis based on the random forest algorithm, as well as the receiver operating characteristic curve (ROC) and the area under the curve (AUC), was performed following the Rpubs documentation (https://rpubs.com/jigbadouin/RFEA1130, accessed on 12 November 2024). For missing values imputation MICE package (Multiple Imputation by Chained Equations) using the predictive mean machine (ppm) method was used.

## 3. Results

### 3.1. Data

Having evaluated the reproducibility of the metabolites, as well as the chromatographic performance and detection methods, 211 human fecal samples were analyzed. The QCs, both validation and calibration, were used to check analytical reproducibility. Once data quality was verified, normalized, and trend corrected, 127 metabolic features were detected and included in the subsequent multivariate and univariate data analysis.

### 3.2. Cohort Description

Once the quality of the analysis was checked, several observations were considered for further statistics. Age, fecal Hb amount, and gender were studied to determine if these variables were significantly different among groups (Table 1). In the case of fecal Hb, both numerical and categorical variables were tested. The PSPP tool was used to perform the one-way ANOVA. As can be observed from Table 1, more detailed information is gathered in Tables S1–S4; age, gender, and fecal Hb were significantly different among groups. The ANOVA *p*-values for all variables were <0.05. As the Levene test for homogeneity of variance was significant in all cases, a multiple comparison post hoc test based on Games–Howell was performed to determine where the differences lied. For age (Table S1), significant *p*-values were obtained between CRC and CTRL (*p*-value < 0.001) as well as AA and CTRL (*p*-value = 0.001). In the case of gender (Table S2), significant *p*-values were obtained only between CRC and CTRL with a *p*-value of 0.024. For fecal Hb (Tables S3 and S4), significant *p*-values were found in all comparisons for both fecal Hb treated as a categorical variable and as a numerical variable. Thus, all factors were considered for further statistical analysis.

### 3.3. Statistical Analysis

For the multivariate analysis, an overall analysis of the samples was conducted by using PCA analysis and including all fecal samples as well as QCs, both calibration QC and validation QC, were performed. These QCs were reference plasma samples (calibration QC), extracted and analyzed at the same time as the individual samples, as well as a pool from all the fecal samples (validation QC). Both QC samples were included in this first PCA analysis to assess the quality of the analysis as well as the normalization and correction process. The proximity among the QCs observed in the scores plot of this PCA (Figure S1) indicated a good reproducibility and quality of measurements.

**Figure 1:**
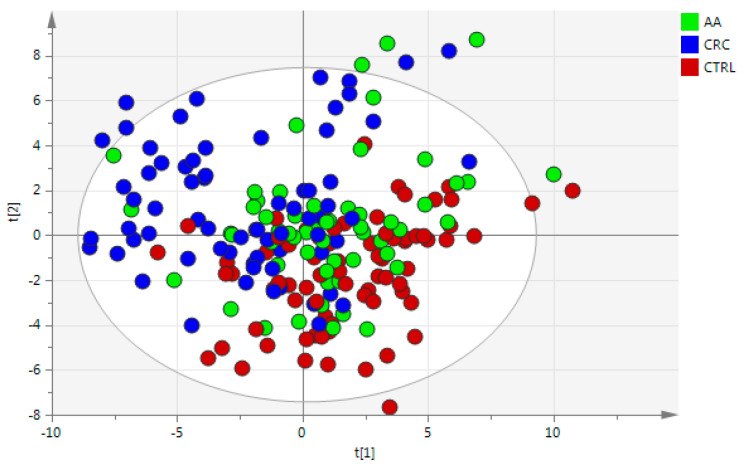
Scores plot of PLS-DA model of fecal samples (class label = group CRC, AA, and CTRL). Autoscaling and logarithm transformation of the data. R2X = 0.465, R2Y = 0.262, Q2 = 0.155, and 3PCs. CV-ANOVA *p*-value = 2.88 × 10^−17^.

Due to the lack of homogeneity in terms of age and fecal Hb amount previously found between groups by one-way ANOVA (Tables S1 and S3), both variables were included in multivariate analysis. Data of these variables were standardized to a median of 0 and a standard deviation of 1 to reduce the influence of differences in scales. Then, autoscaling and logarithm transformation were used to transform the non-normal data into data that follows a normal distribution, followed by the procedure proposed by George Box and David Cox [21]. As can be observed in the PCA (Figure S1), none of the components shows a separation among groups. Some samples were identified as potential outliers since they were outside the Hotelling’s T2 ellipse (95% confidence interval) and they were removed from further multivariate statistical analysis (highlighted red dots in Figure S2). Afterwards, a PLS-DA using groups (CRC, AA, and CTRL) as classification labels was built to enhance the separation. The validated PLS-DA model (see Figure 1) with a CV-ANOVA *p*-value of 2.88 × 10^−17^ showed a tendency of separation among CRC, AA, and CTRL samples. As can be observed from this figure, the AA group is completely mixed with the remaining CRC and CTRL samples. Finally, gender was tested as a classification label, but no PLS-DA model was obtained, indicating that gender did not separate samples into groups.

**Figure 2:**
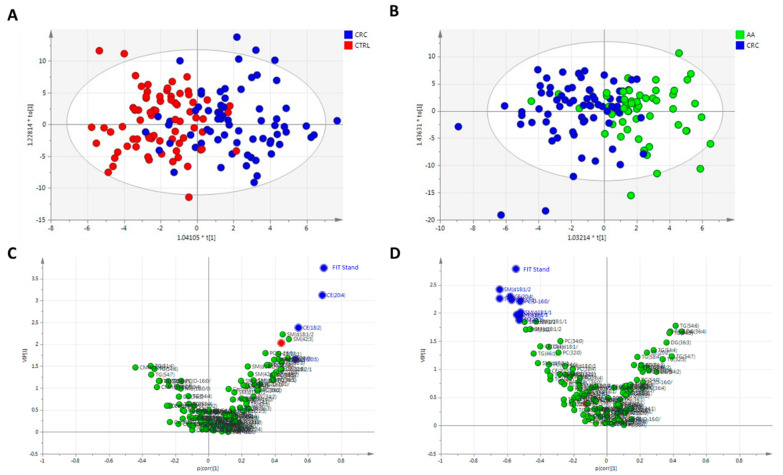
Two-by-two comparisons based on OPLS-DA models. (**A**) Scores plot of CRC vs. CTRL individuals. Autoscaling and logarithm transformation of the data. R2X = 0.215, R2Y = 0.524, Q2 = 0.385, and 2PCs. CV-ANOVA *p*-value = 1.22 × 10^−12^. (**B**) Scores plot of CRC vs. AA patients. Autoscaling and logarithm transformation of the data. R2X = 0.473, R2Y = 0.420, Q2 = 0.215, and 3PCs. CV-ANOVA *p*-value = 2.63 × 10^−4^. (**C**) Volcano plot of CRC vs. CTRL individuals; highlighted in blue the important variables with VIP > 1 (*Y*-axis) and |*p*(corr)| > 0.5 (*X*-axis), and red the age stand. (**D**) Volcano plot of CRC vs. AA individuals; highlighted in blue the important variables with VIP > 1 (*Y*-axis) and |*p*(corr)| > 0.5 (*X*-axis), and red the age stand.

In order to properly select variables that influence group separation, two-by-two comparisons based on OPLS-DA were performed. In the case of AA vs. CTRL, no OPLS-DA model was obtained and thus it was not included. The OPLS-DA models that compared CRC vs. CTRL (Figure 2A) and CRC vs. AA (Figure 2B) were validated, with CV-ANOVA *p*-value of 1.22 × 10^−12^ and 2.63 × 10^−4^, respectively. In both cases, a separation was observed, and variables were selected based on the criterion mentioned before (VIP > 1, in *Y*-axis, and |*p*(corr)| > 0.5, in *X*-axis). In the first comparison (CRC vs. CTRL), VIP and *p*(corr) values for standardized age were 2.024 and 0.438, respectively. Similarly, in the comparison of CRC vs. AA, 0.390 for VIP and −0.119 for *p*(corr) were obtained for standardized age. This was indicative that even though age was significantly different based on one-way ANOVA between some groups, age did not influence group separation, and it was not an important variable for CRC diagnosis. Thus, age was not considered as a covariable in further statistical analysis. In the case of fecal Hb amount (FIT stand), this variable was obtained as the most significant and important variable in the separation of CRC from the other two groups, AA and CTRL, and further studies were performed. In total, 4 analytes were upregulated in CRC compared to CTRL, and 10 were upregulated in CRC compared to AA patients with VIP values greater than 1 and absolute values of *p*(corr) greater than 0.5 (Table 2; Figure 2C,D).

Normal distribution was also studied in the complete cohort and per group. Univariate normality, assessed by Shapiro–Wilk test, pointed out that most of the metabolites did not follow a normal distribution, as can be observed in the Supplementary Excel File S1. Due to this, univariate statistical results were based on non-parametric tests: robust fold-changes (fold-change of the medians), and the Wilcoxon–Mann–Whitney test (*p*-value). Finally, a multiple hypothesis correction test at a level of α = 0.05 was controlled by Benjamini–Hochberg correction test (*q*-value). All this information was gathered in the Supplementary Excel File S1. To reduce and simplify the interpretation, univariate statistical analysis was summarized as heatmaps showing the results of the comparisons AA vs. CTRL, CRC vs. CTRL, and AA vs. CRC (Supplementary Excel File S2).

As can be observed from the heatmap (Supplementary Excel File S2), the differences in the metabolites of the AA group compared with the CTRL group were not significant (*q*-value > 0.05), even some differences were obtained in the log2 (robust fold change). The biggest differences were mainly observed in the cholesteryl ester (CE) lipid class, such as CE (20:4), CE (22:6), and CE (20:5), where the abundances were increased in AA group (positive log2(robust fold-change)), and also in the TG (51:4) and Cer (d18:1/18:0), with decreased levels in AA (negative log2(robust fold-change)) (see Supplementary Excel File S2). On the other hand, the CRC group presents significant differences in several classes of lipids when compared to the other two groups of patients, such as diglycerides (DGs) and triglycerides (TGs), CEs, phosphatidylcholines (PCs), and sphingomyelins (SMs) (Supplementary Excel File Supplementary Excel File S2). Table 3 and Table 4 show a summary of the univariate statistical analysis results based on log2 (robust fold-change) and *q*-values of significant metabolites. In total, 28 and 26 metabolites were significant between CRC vs. CTRL (Table 3) and CRC vs. AA (Table 4), respectively.

As it was previously mentioned, fecal Hb variable (Tables S3 and S4) was different between groups, and it was the variable that most influenced group separation when CRC was compared with CTRL or AA (Figure 2C,D). However, despite this fact, FIT was considered a not very sensitive tool for the diagnosis [13]. A positive fecal Hb result during the screening was considered when levels of haemoglobin in stool were greater than 20 µg Hb/g feces. In this study, the percentage of hits for CTRL individuals, which means negative results for the FIT screening test, was 73.08%; in the case of AA and CRC, which means positive results, were 50.67% for AA and 86.67% for CRC. All results, being positive or negative, were confirmed by colonoscopy. It can be pointed out from the results of this cohort that FIT screening tests, as a unique diagnosis measurement, might be sensitive for CTRL and CRC individuals, where 21 out of 78 CTRL individuals obtained a false positive result, and 10 out of 75 of CRC patients obtained a false negative result, respectively. However, in the case of AA, 20 out of 58 were classified as negative results for fecal Hb.

In order to determine the accuracy of FIT as well as the power of CEs to enhance colorectal cancer stage diagnosis, ROC and AUC were calculated as evaluation metrics for the classification algorithm of random forest. In this sense, 70% of the data was used as a training set to set up the predictive model and the remaining 30% was used as a test set to determine the predictive power. The CEs included were those determined with the lipidomics analysis: cholesterol and derivatives, CE(18:1), CE(18:2), CE(20:2), CE(20:4), CE(20:5), CE(22:4), CE(22:5), and CE(22:6).

Prediction model together with ROC AUC metrics that quantify the ability of a binary classifier to distinguish two classes are gathered in Table 5. The out-of-bag (OOB) error is a method of measuring the prediction error of the random forest and provides an accurate estimate of the model’s performance using the subset of training samples that were not used (out-of-bag samples) in creating the decision tree. Taking this into account, the accuracy was greater, and the OOB estimated error was less when all CEs were included in the model, except for the comparison AA vs. CTRL, and CTRL vs. AA + CRC. The findings in this last comparison were probably due to the effect of AA samples that influence the lack of predictive power. This might be because the AA stage can be considered a transitionary stage between CTRL and CRC individuals, and thus, some samples were closer to CTRL and others to CRC phenotype. As accuracy can be influenced by class-imbalanced datasets, leading to an overestimation biased toward the majority class, other metrics should be considered, such as precision, recall, and F1-score (Table 5), to correctly validate the model. To quickly review, precision is the proportion of true positives among all predicted positive cases, recall is the proportion of true positives among all actual positive cases, and F1-score is a single metric that combines recall and precision to gain a better understanding of model performance and indicates the reliability of the model. Considering all these parameters, models CRC vs. CTRL and CRC vs. AA containing fecal Hb, as well as CEs, gave a better result than those containing only fecal Hb. However, in the cases of CTRL vs. AA only or AA + CRC, no improvement was obtained by adding CEs into the models. Finally, the AUC of the ROC curves showed the same behaviour as the other metrics used for model quality evaluation. Thus, adding CEs to the model, the predictive power was improved for the comparisons that include CRC patients with excellent results for AUC value, but for those with AA samples, the results were practically the same, being some metrics better and others worse when adding CEs, but with acceptable AUC values. This demonstrated that CEs were valuable metabolites to better diagnose CRC and distinguished them from both CTRL and AA, decreasing the errors associated with CRC diagnosis, even though such improvement was not observed for any comparison that included AA.

**Figure 3:**
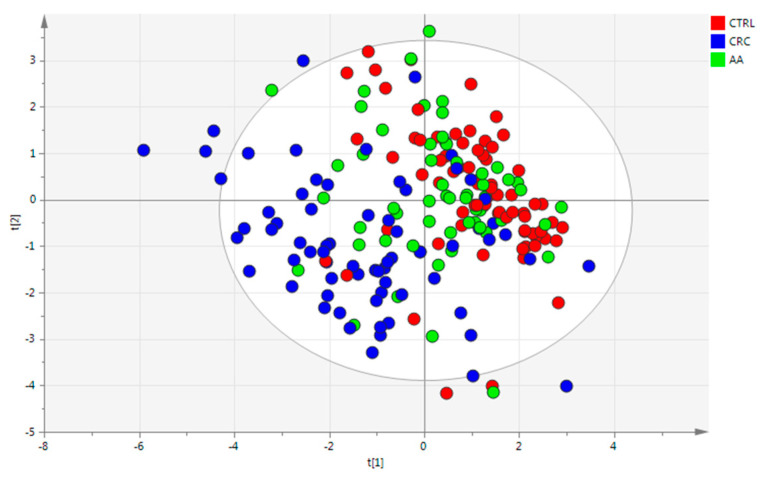
Scores plot of PLS-DA model of fecal samples (class label = group—CRC, AA, and CTRL) using CEs and FIT stand as variables. Autoscaling and logarithm transformation of the data. R2X = 0.614, R2Y = 0.221, Q2 = 0.188, and 2PCs. CV-ANOVA *p*-value = 7.49 × 10^−27^.

To see the improvement on diagnosis using FIT as well as CEs, PLS-DA (Figure 3) and two-by-two OPLS-DA models (data not included) were generated as previously mentioned, but only containing the FIT stand and the CEs. In the case of the PLS-DA scores plot (Figure 3), a clear tendency of CRC separation compared to the remaining two groups was found; however, AA samples were still mixed mainly with CTRL. What was of special significance was that some AA samples were more closely associated with the CRC group and some others more with the CTRL group. This was in agreement and might explain what was also found by random forest when AA and CRC were considered as unique groups. Thus, the findings support the hypothesis previously observed, where some AA samples had profiles or phenotypes more like CRC samples, which might explain a more advanced transitionary stage from AA to CRC.

The classification list obtained for PLS-DA that provides the samples as well as the original and the predicted values for each group (CRC, AA, or CTRL) was further analyzed. The values displayed in the classification list were original values (YvarPS) and predicted values (YpredPS) (see Supplementary Excel File S3). The observations were coloured such that values <0.35 were white (do not belong to the class), between 0.35 and 0.65 were orange (borderline), and >0.65 were green (belong to the class). The detailed information is gathered in the supplementary table (Supplementary Excel File S3).

Considering the three groups at the same time and analyzing the classification list obtained from the PLS-DA model, only 4 CTRL individuals out of 74 were classified as CRC with values > 0.65 and the other 4 were on the borderline to be classified as CRC individuals. Doing the same but for CRC, only 1 sample out of 69 was misclassified as CTRL (value > 0.65) and 12 were on the borderline to be classified as CTRL or AA. In these two cases, compared with the number of false positives obtained by the FIT parameter for CTRL (21 out of 78) and false negatives for CRC (10 out of 75), the diagnosis was clearly improved by adding CEs, where 70 out of 74 CTRL were correctly labelled. In the case of CRC, 98% of the CRCs were correctly assigned. The highest misclassification ratio was obtained for the AA group, where none of the samples were classified as AA. This was in accordance with the PLS-DA scores plot (Figure 3), where all AA samples were located mainly with CTRL and CRC.

Among the two-by-two comparisons (OPLS-DA), only the comparisons CRC *vs*. CTRL and CRC vs. AA were significant, as was also found previously. Studying the classification lists, it was found that the results are presented in Table 6. CE profile together with fecal Hb correctly classified 91.04% and 96.49% of CTRL individuals compared to CRC or AA, 78.12% and 36.84% of AA compared to CRC or CTRL, and 85.45% and 82.76% of CRC compared to AA and CTRL, respectively. Note that only cases correctly classified (>0.65) or not classified (<0.35) were used for percentages.

The last question proposed in this study, based on the FIT screening diagnostic tool, was how FIT influences separation. For this purpose, age stand, and FIT stand were removed from the multivariate analysis and FIT as categorical variable was included as class label. Similarly to the previous workflow, PCA was first built to see any putative tendency and to detect outliers. Then, a validated PLS-DA model using FIT (yes/no) as class was obtained with a CV-ANOVA *p*-value of 4.64 × 10^−5^ (Figure S3). In this case, the sample group was added as a label to show that even though the model was validated with a separation tendency, no tendency based on CRC, AA, or CTRL groups was found. In Table S5, those variables that fulfilled the VIP and |*p*(corr)| cut-off criteria were included. Most of the metabolites responsible of group separation based on FIT (yes/no) belonged to lipid classes of glycerolipids (DGs and TGs) and sphingolipids (SMs), and just one cholesteryl ester (CE (18:2)). Among them, the CE (18:2) and some SMs were commonly found with the metabolites responsible of group separation when CRC, AA, or CTRL were used as group labels. This was a very interesting finding as the lipids that separated groups based on fecal Hb belonged to DG and TG classes, whereas those responsible for group separation based on disease were mainly PCs, SMs, and CEs.

### 3.4. Cholesteryl Esters as Targets in CRC Studies

In order to compare the results included in this study with previous works, CEs were analyzed and compared between those obtained in fecal samples and/or FIT extracts [11,12]. Among them, CE (18:1), CE (18:2), CE (20:2), CE (20:4), CE (20:5), CE (22:4), CE (22:5), and CE (22:6) were considered. In Table 7, the information related to the log2 (robust fold change) and the *q*-value obtained in this study is gathered.

The comparison of the results obtained in Table 7 with previous studies showed that no differences were obtained between AA and CTRL in any case, and several CEs were significantly different in the other two comparisons (CRC vs. CTRL, and CRC vs. AA) [11,12]. When results of the analysis of fecal samples from previous study was compared with the current results, it was found that CE (18:2) and CE (20:4) were significantly upregulated (*q*-value < 0.05) in CRC compared to CTRL and to AA, with log2 (FC) greater than 1, which mean more than double abundance in CRC. These results and tendencies were also in agreement with the results obtained in FIT extracts, where CE (18:2) was significantly enhanced in CRC compared to AA but not to CTRL, and CE (20:4) was significantly upregulated in CRC in both comparisons. All these studies pointed out the same CEs as the most important or relevant ones using both different types of samples as well as different population cohorts, which supports the robustness and reliability of the results.

In order to study these two CEs, the same Rscript based on random forest algorithm, ROC, and AUC metrics was used but only using the fecal Hb as well as CE (18:2) and CE (20:4). The results were included in Table 8. Reducing the CEs to those commonly found in all studies performed in feces or FIT extract, it was found that for most of the metrics the percentage decrease a bit for the comparisons of CRC vs. CTRL or AA but, what was of special interest was that all metrics improved in the comparison of CTRL vs. AA except recall. This was a very interesting finding as these two CEs enhanced AA classification when compared to CTRL with an AUC of 70% and an estimated error of 36.78%, which was 2.3% less error than only fecal Hb. Thus, it seems that the CEs’ profile enhances the classification and diagnosis of CRC compared to AA or CTRL but only CE (18:2) and CE (20:4) were needed to improve the classification of AA compared to CTRL. Despite the decrease in the other two remaining comparisons with only two CEs, the diagnosis classification and predictive metrics were acceptable and better than FIT alone. It was also observed that AA and CRC, as the same group, decrease the predictive metrics and the accuracy of these models, probably due to both stages being more differentiated in terms of metabolism than in FIT.

## 4. Discussion

The mechanisms underlying the pathogenesis, progression, and disparities of CRC have not been completely understood but it is believed that many factors are involved. Among them, lipid metabolism alterations have been pointed out as important risk factors associated with CRC. Most cases of CRC occur spontaneously, where half of the patients who undergo surgery survive, but overall survival rates are decreased by late diagnosis [6]. Thus, an accurate early diagnosis tool is required. The current screening tool, based on the measurement of Hb in feces (FIT test), has demonstrated efficacy in randomized trials; however, data collected of FIT from different population cohorts have pointed out that FIT has low sensitivity for AA, where the diagnostic error was bigger than for healthy individuals or CRC patients. In the present study, we have performed a UHPLC-based metabolomics analysis of stool to detect endogenous metabolites that could improve the early diagnosis of CRC and/or AA patients. As CEs are the most representative lipid class that was significantly obtained for group separation in this study, they are further studied as potential diagnostic markers of CRC and AA patients. Furthermore, CEs are commonly found to be significant among several studies [11,12] and thus, they are considered the most relevant diagnostic markers.

The metabolic pathways that regulate the synthesis and hydrolysis of CEs are critical to maintain the balance of intracellular free cholesterol and protect from its toxicity. Free cholesterol is an essential constituent of the cell membrane, plays a key role in cell survival and proliferation, and serves as a precursor of important materials in all living cells, such as bile acids, steroid hormones, and oxysterols [22,23]. Therefore, the maintenance of cholesterol homeostasis is crucial for physiological functions. The dysregulation of such homeostasis not only leads to cardiovascular diseases, but also is involved in tumorigenesis and progression of cancer [23]. Cholesterol homeostasis requires ensuring sufficient cholesterol biosynthesis and uptake for cell growth and function, while also preventing the overabundance of intracellular cholesterol through esterification, efflux, and processing. When intracellular cholesterol levels exceed demand, excess cholesterol can be esterified to CEs by acyl CoA:cholesterol acyltransferases (ACATs) and stored in the cytoplasm in lipid droplets [24,25]. The dysregulation of this homeostasis is a characteristic feature of cancer cells due to the requirements of high cholesterol levels for membrane formation and signal transduction because of their rapid proliferation compared to normal cells [26,27]. To maintain that rapid proliferation and progression, cancer cells greatly increase demands for free cholesterol [28], which can be obtained by up-taking from the extracellular environment (absorption of cholesterol) or from intracellular synthesis de novo. Also, and importantly, cholesterol supplementation can be obtained from hydrolysing the CEs largely stored in lipid droplets of cancer cells by the action of cholesterol ester hydrolase (CEH) and lysosomal acid lipase (LAL) [24,29]. Several studies have pointed out that CEs metabolism highly correlates with the pathogenesis and progression of many cancers [24,30,31] due to the ability of cancer cells to reprogram cholesterol metabolism to maintain continuous proliferation. It is proposed that the hydrolysis of CEs can be the fastest and most cost-effective way to obtain the needed free cholesterol [24]. Among all previous enzymes mentioned, it has been reported that tumours exhibit high expression of ACAT1 [23,24,31,32,33], one of the isoforms of ACAT, accompanied by elevated levels of CEs, indicating that ACAT1 is crucial for cancer cells to regulate cholesterol metabolic homeostasis [34]. The process by which ACAT1 converts cholesterol into CE occurs in many types of aggressive cancers and catalyses the reaction between long-chain fatty acyl-CoA (saturated or unsaturated fatty acids containing 13–21 carbons) and intracellular cholesterol [35].

A study performed in prostate cancer cells demonstrated that the inhibition of ACAT1 significantly reduces the CE level [31]. They found that prominent CE accumulation, which only occurred in advanced prostate cancer, is a consequence of the loss of tumour suppressor PTEN and subsequent activation of the PI3K/AKT/mTOR pathway. PTEN regulates metabolic pathways to meet the demand of aggressive prostate cancer cells for LDL cholesterol uptake. Also, Wu et al. found that cholesterol activates the PI3K/AKT pathway to promote CRC progression [36] as well as in other human cancers [37,38]. Initially, Yue et al. suspected that CE storage might act as a pool for fatty acid and cholesterol, which can be released from lipid droplets for cancer cell proliferation [31]. However, they noticed that low-density lipoprotein (LDL), which is the way most cells absorb cholesterol via LDL-mediated endocytosis [39], is the primary carrier of essential polyunsaturated fatty acids, including arachidonic acid (FA 20:4) [31]. In this sense, the LDL uptake was linked to arachidonic acid, which is released from LDL and converted to a range of eicosanoids that have been pointed out to be implicated in various pathological processes, including inflammation and cancer [31]. This study also showed that depleting CE storage disrupts intracellular cholesterol homeostasis and consequently reduces the uptake of essential fatty acids such as arachidonic acid, which is a proliferation factor of prostate cancer [40,41]. Depleting CEs would imply that free cholesterol levels increase, and apoptosis induced by ACAT inhibition could occur due to the toxicity of free cholesterol. Yue S. et al. also found that CE-depleting drugs do not cause detectable toxicity to normal cells and thus, cholesterol esterification might be a cancer-specific target for cancer therapy [31]. This approach is used to treat cells of different diseases, such as lymphocytic leukaemia [42], glioblastoma [43], and breast cancer [44]. All this information highlights the importance of CE (20:4) that could explain its role in the progression of CRC. The difference between CRC vs. CTRL based on log2 (robust fold-change) is higher than between CRC vs. AA, which could be in accordance with the progression of cancer as shown with prostate cancer cells [31,40,41]. As more amount of CE (20:4) more amount of arachidonic acid can be released and consequently increase the proliferation of cancer cells.

Once the biological role as well as the mechanisms behind CE synthesis and hydrolysis have been established, our final goal is to generate a hypothesis that could explain the presence of free CEs in fecal samples. Shortly, CEs must first be hydrolyzed before cholesterol can be absorbed in the small intestine. The enzyme cholesterol esterase, secreted by the pancreas, catalyses the reaction to maintain homeostasis by controlling the bioavailability of cholesterol obtained from dietary CEs to be absorbed as no esterified cholesterol [45]. This first approach suggests that free CE accumulation might be due to a dysfunction in the cholesterol esterase enzyme or due to malabsorption of dietary nutrients, generally attributed to the consequences of oncologic treatments that reduce the gastrointestinal absorption [46]. It has been reported that malabsorption of nutrients, which is a stage of malnutrition, is associated with worse clinical outcomes in cancer patients [47].

The other approach to explain CEs in fecal samples is related to tumour-derived exosomes that it was reported to be associated with tumour development and metastasis [48]. However, those CEs are not part of the membrane architecture of the exosomes. Despite this fact, it is noted that exosomes can contain cholesterol esters, i.e., CEs are efficiently eliminated from reticulocytes by exosomes during early stages of their maturation [49]. CEs are generated downstream of receptors involved in normal and tumour cell proliferation, such as the cholecystokinin (CCK2) receptor [50]. In this sense, since tumoral cells constitutively release exosomes, these vesicles can potentially deliver CEs into the endosomes of other cells and promote their proliferation [48,50]. The presence of CEs in exosomes obtained from different samples and cell types [51,52,53,54,55], together with the proliferation pathway described previously, enhances this hypothesis of CEs transported by exosomes in colorectal cancer for tumour cell proliferation.

## 5. Conclusions

The results included in this study pointed out that fecal Hb, together with CEs, could be a promising diagnostic tool that improves the accuracy of diagnosis compared to the use of fecal Hb only, mainly for CRC and CTRL. The predictive models have been demonstrated to reduce both false positives and false negatives. Also, the comparisons performed among different studies have highlighted the CE (20:4) as the most significant and relevant CE, together with CE (18:2).

These results are in accordance with other cancer studies in two possible ways. The first one implies that the accumulation of CEs, due to the increased expression of ACAT1, is involved in cholesterol metabolic homeostasis. This approach is in agreement with the cholesterol requirements of cancer cells for membrane formation in a fast and cost-effective way. The second implies that the accumulation of CEs, due to PTEN loss and PI3K/AKT activation, promotes colorectal cancer progression. AKT mediates the activation of mTOR complex 1 that regulates the function of transcription factors (SREBPs). These factors are involved in controlling lipid and cholesterol homeostasis by sending cholesterol through LDL absorption. These findings shed more light on the mechanisms behind the progression of CRC and highlight two putative therapeutic targets. The inhibition of ACAT1 to reduce the accumulation of CEs or the blockage of cholesterol esterification to reduce the absorption of LDL containing arachidonic acid, which is a critical proliferative factor activating the PI3K/AKT pathway.

Finally, two putative hypotheses related to the presence of CEs in fecal samples are proposed. The first relates to a dysfunction in CE hydrolysis to be absorbed as cholesterol in the small intestine or a malabsorption of dietary nutrients and the second relates to CEs transported inside exosomes from cell to cell to promote proliferation. Both hypotheses are in agreement with previous studies and should be further investigated.

## Figures and Tables

**Table 1:** Classification of the patients included in the study. Diagnosis based on colonoscopy. Number of patients with positive (+; fecal haemoglobin concentration ≥ 20 µg Hb/g feces) or negative (−) results in the fecal immunochemical test (FIT).

	Diagnosis			ANOVA *p*-Value
	Control (CTRL)	Adenoma (AA)	Colorectal Cancer (CRC)	
N	78	58	75	Not applicable
Gender (% women)	50	34	29	0.024
Age (years; mean ± sd)	68.40 ± 14.85	76.69 ± 9.95	77.81 ± 10.74	<0.001
FIT (+)	21	38	65	<0.001
FIT (−)	57	20	10	

**Table 2:** Variables selected from OPLS-DA models with the corresponding VIP and *p*(corr) values when CRC, AA, and CTRL were used as classification labels.

	Variable	*p* (corr)	VIP ^a^
CRC vs. CTRL	Standardized fecal Hb	0.6948	3.7443
	CE (20:4)	0.6846	3.1189
	CE (18:2)	0.5408	2.3798
	CE (20:5)	0.5250	1.6608
CRC vs. AA	Standardized fecal Hb	−0.5484	2.7858
	SM (d18:1/24:1) + SM (d18:2/24:0) ^b^	−0.6451	2.4180
	CE (20:4)	−0.5825	2.2940
	SM (d18:2/24:1) + SM (d18:1/24:2) ^b^	−0.6459	2.2555
	SM (d17:1/16:0) + SM (d18:1/15:0) ^b^	−0.5728	2.2294
	PC (O-16:0/16:0)	−0.5203	2.2089
	SM (d18:1/16:0)	−0.5186	2.0138
	SM (d18:1/17:0)	−0.5421	1.9714
	CE (18:2)	−0.5229	1.9402
	SM (42:1)	−0.5280	1.8786

^a^ Variable importance on projection; ^b^ chromatographic overlap of two or more isomeric metabolites.

**Table 3:** Metabolites selected from univariate statistical analysis in the comparison of CRC vs. CTRL with the corresponding log2(robust fold-change) and *q*-value.

Metabolite	Log2(Robust Fold-Change)	*q*-Value
CE (18:1)	0.7931	5.70 × 10^−3^
CE (18:2)	1.8460	4.10 × 10^−6^
CE (20:4)	2.5633	7.70 × 10^−10^
CE (20:5)	1.2566	1.50 × 10^−2^
CE (22:6)	1.0574	4.70 × 10^−3^
PC (16:0/16:0)	0.6417	6.50 × 10^−3^
PC (16:0/16:1) + PC (14:1/18:0) + PC (14:0/18:1) ^b^	0.8143	5.90 × 10^−3^
PC (16:0/18:1)	0.7270	1.10 × 10^−2^
PC (18:0/18:1)	0.7239	1.80 × 10^−2^
PC (16:0/20:4)	0.4835	1.50 × 10^−2^
PC (18:0/20:4)	0.4735	7.40 × 10^−3^
PC (16:0/22:6)	1.1738	4.40 × 10^−3^
DG (32:1)	0.4271	4.50 × 10^−2^
PC (O-16:0/16:0)	0.6236	8.20 × 10^−3^
PC (O-16:0/18:1) + PC (18:1e/16:0) ^b^	0.8781	1.60 × 10^−2^
PC (O-16:0/18:2)	1.1600	7.40 × 10^−3^
PC (O-18:0/18:2)	0.5693	4.80 × 10^−2^
SM (d17:1/16:0) + SM (d18:1/15:0) ^b^	0.9500	7.40 × 10^−3^
SM (d18:1/16:0)	0.9900	5.70 × 10^−3^
SM (d18:2/16:0)	0.7225	8.00 × 10^−3^
SM (d18:1/23:0)	1.1646	3.90 × 10^−2^
SM (d18:1/24:1) + SM (d18:2/24:0)	1.3371	1.20 × 10^−3^
SM (d18:2/24:1) + SM (d18:1/24:2) ^b^	0.9671	1.40 × 10^−2^
SM (42:1)	0.8341	3.50 × 10^−2^
TG (18:2_18:2_15:0) + TG (17:1_18:2_16:1) + TG (17:1_18:3_16:0) ^b^	−1.0903	5.90 × 10^−3^
TG (16:0_18:2_18:3) ^b^	−1.3093	4.50 × 10^−2^
TG (18:2_18:3_18:1) ^b^	−1.8290	2.10 × 10^−2^
TG (54:7)	−1.7835	4.50 × 10^−2^

^b^ Chromatographic overlap of two or more isomeric metabolites.

**Table 4:** Metabolites selected from univariate statistical analysis in the comparison of CRC vs. AA with the corresponding log2(robust fold-change) and *q*-value.

Metabolites	Log2(Robust Fold-Change)	*q*-Value
CE (18:1)	0.6281	5.00 × 10^−2^
CE (18:2)	1.4507	1.70 × 10^−3^
CE (20:2)	−0.7016	5.00 × 10^−2^
CE (20:4)	1.8026	1.90 × 10^−3^
PC (16:0/16:0)	0.5055	4.40 × 10^−2^
PC (16:0/18:0)	0.1995	2.00 × 10^−2^
DG (18:1_18:2_0:0) ^b^	−1.4547	2.60 × 10^−2^
DG (18:2_18:2_0:0) ^b^	−1.5312	1.50 × 10^−2^
PC (O-16:0/16:0)	0.9914	1.90 × 10^−3^
PC (O-16:0/18:1) + PC (18:1e/16:0) ^b^	0.8829	4.30 × 10^−2^
PC (O-16:0/18:2)	0.9022	1.90 × 10^−2^
SM (d17:1/16:0) + SM (d18:1/15:0) ^b^	0.9711	2.60 × 10^−3^
SM (d18:1/16:0)	1.2231	4.30 × 10^−3^
SM (d18:1/17:0)	1.0336	3.50 × 10^−2^
SM (d18:1/18:0)	1.6791	5.70 × 10^−3^
SM (d18:1/18:1) + SM (d18:2/18:0) b	1.1187	2.60 × 10^−2^
SM (d18:1/22:0)	1.3192	1.80 × 10^−2^
SM (d18:1/23:0)	1.3881	1.40 × 10^−2^
SM (d18:1/24:1) + SM (d18:2/24:0)	1.7776	1.70 × 10^−3^
SM (d18:2/24:1) + SM (d18:1/24:2) ^b^	0.9900	2.60 × 10^−2^
SM (42:1)	1.1880	1.40 × 10^−2^
TG (16:0_18:2_18:2) ^b^	−1.4691	2.00 × 10^−2^
TG (18:2_18:1_18:1) + TG (18:2_18:2_18:0) ^b^	−1.4075	4.40 × 10^−2^
TG (18:2_18:2_18:1) ^b^	−1.6763	1.40 × 10^−2^
TG (18:2_18:3_18:1) ^b^	−1.8445	3.30 × 10^−3^
TG (18:2_18:3_18:2) ^b^	−1.1997	3.60 × 10^−2^

^b^ Chromatographic overlap of two or more isomeric metabolites.

**Table 5:** ROC AUC metrics obtained for the test set for FIT or the combination of FIT with the CEs in a random forest classification model for the comparisons CRC vs. CTRL, CRC vs. AA, and AA vs. CTRL.

2-by-2 Comparison	Variable(s)	OOB Estimate Error Rate (%)	Accuracy	Precision	Recall	F1-Score	AUC
CRC vs. CTRL	FIT	22.45	83.64	85.71	82.76	84.21	90
	FIT + CEs	19.39	89.09	89.65	89.65	89.65	91
CRC vs. AA	FIT	33.72	63.83	77.78	65.62	71.19	69
	FIT + CEs	32.56	74.47	75.00	93.75	83.33	81
AA vs. CTRL	FIT	39.08	69.39	69.05	93.55	79.45	76
	FIT + CEs	45.98	65.31	64.58	100	78.48	70
CTRL vs. AA + CTRL	FIT	32.33	82.05	78.12	78.12	78.12	84
	FIT + CEs	27.07	74.36	63.04	90.62	74.36	84

**Table 6:** Summary of the classification list results obtained for validated OPLS-DA models using only FIT stand and CEs.

		Samples Correctly Classified (YpredPS > 0.65)	Samples in the Borderline (0.35 > YpredPS < 0.65) ^c^	Samples Not Classified (YpredPS < 0.35)
CRC vs. CTRL	CRC	48	11 (4)	10
	CTRL	61	7 (1)	6
CRC vs. AA	CRC	47	14 (3)	8
	AA	25	24 (7)	7
CTRL vs. AA	CTRL	32	37 (13)	5
	AA	7	37 (5)	12

^c^ In brackets, it contained the number of samples with values > 0.6.

**Table 7:** Fecal levels of the nine metabolites of interest between patients with AA and CRC vs. CTRL individuals, and CRC vs. AA.

	AA vs. CTRL		CRC vs. CTRL		CRC vs. AA	
Metabolites	log2 (Robust FC)	*q*-Value	log2 (Robust FC)	*q*-Value	log2 (Robust FC)	*q*-Value
Cholesterol and derivatives	−0.045	0.9201	0.012	0.6467	0.058	0.8154
CE (18:1)	0.165	0.8436	0.793	0.0057	0.628	0.0498
CE (18:2)	0.395	0.8436	1.846	4.10 × 10^−6^	1.451	0.0017
CE (20:2)	0.383	0.8436	−0.319	0.1909	−0.702	0.0498
CE (20:4)	0.761	0.1739	2.563	7.72 × 10^−10^	1.803	0.0019
CE (20:5)	0.809	0.8436	1.257	0.0154	0.448	0.3486
CE (22:4)	0.054	0.9201	−0.052	0.9551	−0.106	0.8732
CE (22:5)	0.212	0.8436	−0.136	0.8465	−0.348	0.6582
CE (22:6)	0.783	0.7589	1.057	0.0047	0.275	0.5992

**Table 8:** ROC AUC metrics obtained for the test set for the predictive random forest model that includes FIT and CE (18:2) and CE (20:4) for the comparisons CRC vs. CTRL, CRC vs. AA, and AA vs. CTRL.

2-by-2 Comparison	OOB Estimate Error Rate (%)	Accuracy	Precision	Recall	F1-Score	AUC
CRC vs. CTRL	21.43	89.09	89.65	89.65	89.65	89
CRC vs. AA	29.07	68.08	69.77	93.75	80.00	79
AA vs. CTRL	36.78	71.43	71.80	90.32	80.00	70
CTRL vs. AA + CRC	31.58	71.79	62.50	78.12	69.44	80

## Data Availability

The LC-MS metabolomics data for this study are available at the NIH Common Fund’s National Metabolomics Data Repository (NMDR) website, the Metabolomics Workbench [56], https://www.metabolomicsworkbrnvh.org, where it has been assigned Study ID ST003798. The data can be accessed directly via its project DOI: http://dx.doi.org/10.21228/M8WR76 (accessed on 18 March 2025).

## Supplementary Materials

The following supporting information can be downloaded at https://www.mdpi.com/article/10.3390/cancers17142339/s1, Figure S1: Scores plot of the PCA model of fecal and QC samples. Logarithm transformation of the data. R2X = 0.693, Q2 = 0.683, 2PCs. The ellipse represents 95% confidence interval according to Hotelling’s T2 test; Figure S2: Scores plot of the PCA of fecal samples. Autoscaling and logarithm transformation of the data. R2X = 0.605, Q2 = 0.546, 4PCs. Highlighted red dots (samples owl-2323-001, owl-2323-016, owl-2323-019, owl-2323-034, owl-2323-067, owl-2323-068, owl-2323-071, owl-2323-105, owl-2323-109, owl-2323-127, owl-2323-148 and owl-2323-151) were outliers out of the Hotelling’s T2 ellipse; Figure S3: Scores plot of PLS-DA model of fecal samples (class label = FIT–YES (≥20 µg Hb/g feces) and NO (<20 µg Hb/g feces). Autoscaling and logarithm transformation of the data. R2X = 0.465, R2Y = 0.330, Q2 = 0.155, and 3PCs. CV-ANOVA *p*-value = 4.64 × 10^−5^; Table S1: One-way ANOVA and post hoc analysis for age; Table S2: One-way ANOVA and post hoc analysis for gender; Table S3: One-way ANOVA and post hoc analysis for fecal Hb amount in µg/g (numerical variable); Table S4: One-way ANOVA and post hoc analysis for fecal Hb result (categorical variable–yes/no); Table S5: Metabolites selected from PLS-DA models with the corresponding VIP and p(corr) values when FIT (yes/no) was used as classification label; Excel File S1: MX_SuppTable.xlsx. Excel file containing DataMat, which includes the metadata with sample information, and normalized abundances of each metabolite, and data per metabolite, which contains the univariate statistical analysis; Excel File S2: MX_Heatmap.xlsx. Excel file that contains *p*-value and *q*-value as well as log2 (robust fold changes) for all metabolites; Excel File S3: MX_ClassificationList.xlsx. Excel file that includes the original and predicted values for each sample in all predictive models.
