# Supplementary material for: Metabolic Signature in Combination with Fecal Immunochemical Test as a Non-Invasive Tool for Advanced Colorectal Neoplasia Diagnosis
Source: Cancers (Basel). 2025 Jul 15;17(14):2339. doi: 10.3390/cancers17142339 (PMC12293502; doi:10.3390/cancers17142339)
Supplement: Supplementary file 1 [file cancers-17-02339-s001.zip › cancers-3688475-supplementary.pdf]

Supplementary Materials

Metabolic Signature in Combination with Fecal Immunochemical Test as a Non-Invasive Tool for Advanced Colorectal Neoplasia Diagnosis

Oihane E. Albóniga, Joaquín Cubiella, Luis Bujanda, Patricia Aspichueta, María Encarnación Blanco, Borja Lanza, Cristina Alonso and Juan Manuel Falcón-Pérez

Table of content

Page	Description
S4	**Figure S1.** Scores plot of the PCA model of fecal and QC samples. Logarithm transformation of the data. R2X = 0.693, Q2 = 0.683, 2PCs. The ellipse represents 95% confidence interval according to Hotelling's T2 test.
S5	**Table S1.** One-way ANOVA and post-hoc analysis for age.
S6	**Table S2**. One-way ANOVA and post-hoc analysis for gender.
S7	**Table S3**. One-way ANOVA and post-hoc analysis for fecal Hb amount in µg/g (numerical variable).
S8	**Table S4.** One-way ANOVA and post-hoc analysis for fecal Hb result (categorical variable – yes/no).
S9	**Figure S2.** Scores plot of the PCA of fecal samples. Autoscaling and logarithm transformation of the data. R2X = 0.605, Q2 = 0.546, 4PCs. Highlighted red dots (samples owl-2323-001, owl-2323-016, owl-2323-019, owl-2323-034, owl-2323-067, owl-2323-068, owl-2323-071, owl-2323-105, owl-2323-109, owl-2323-127, owl-2323-148 and owl-2323-151) were outliers out of the Hotelling's T2 ellipse.
S10	**Figure S3. Scores plot of PLS-DA model of fecal samples (class label = FIT – YES (≥ 20 µg Hb/g feces) and NO (< 20 µg Hb/g feces).** Autoscaling and logarithm transformation of the data. R2X = 0.465, R2Y = 0.330, Q2 = 0.155, and 3PCs. CV-ANOVA *p*-value = 4.64e-5
S11-S12	**Table S5**. Metabolites selected from PLS-DA models with the corresponding VIP and p(corr) values when FIT (yes/no) was used as classification label
Supplemental File S1	**MX_SuppTable.xlsx. Excel file containing DataMat** which include the metadata with sample information, and normalized abundances of each metabolite, and **Data per metabolite** which contains the univariate statistical analysis.
Supplemental File S2	**MX_Heatmap.xlsx.** Excel file that contains p-value and q-value as well as log2 (robust fold changes) for all metabolites.
Supplemental File S3	**MX_ClassificationList.xlsx.** Excel file which includes the original and predicted values for each sample in all predictive models.

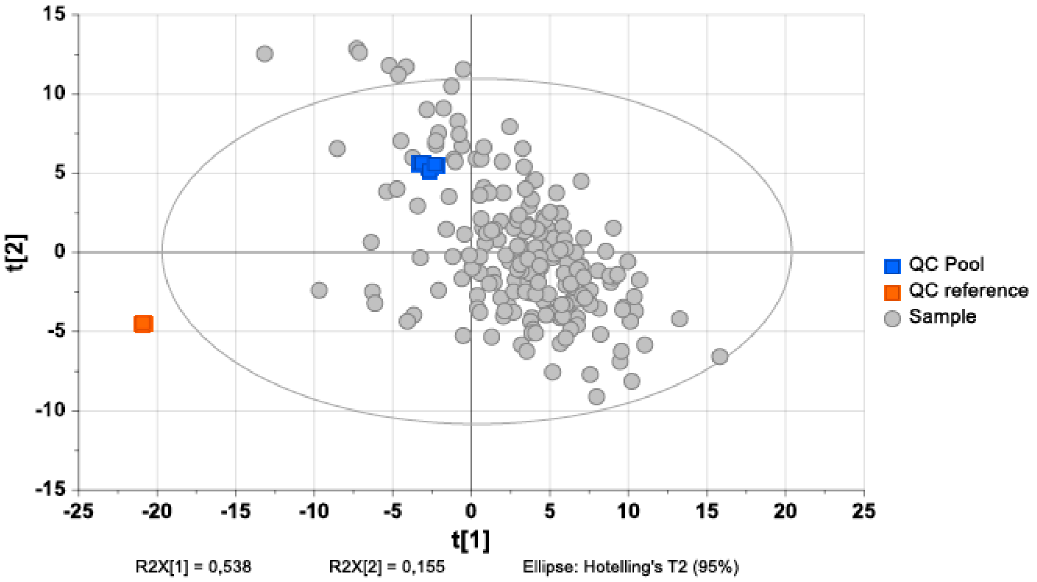

Figure S1. Scores plot of the PCA model of fecal and QC samples. Logarithm transformation of the data. R2X = 0.693, Q2 = 0.683, 2PCs. The ellipse represents 95% confidence interval according to Hotelling's T2 test.

Table S1. One-way ANOVA and post-hoc analysis for age.

Descriptives

	Group	N	Mean	Std. Deviation	Std. Error	95% Confidence Interval for Mean		Minimum	Maximum
						Lower Bound	Upper Bound		
Age	CRC	75	77.81	10.81	1.25	75.33	80.30	50.00	94.00
	AA	58	76.69	10.04	1.32	74.05	79.33	51.00	93.00
	CTRL	78	68.40	14.94	1.69	65.03	71.77	35.00	97.00
	Total	211	74.02	13.01	.90	72.26	75.79	35.00	97.00

Test of Homogeneity of Variances

	Levene Statistic	df1	df2	Sig.
Age	12.21	2	208	.000

ANOVA

		Sum of Squares	df	Mean Square	F	Sig.
Age	Between Groups	3958.40	2	1979.20	13.03	.000
	Within Groups	31590.48	208	151.88		
	Total	35548.88	210			

Multiple Comparisons (Age)

	(J) Family	(J) Family	Mean Difference (I - J)	Std. Error	Sig.	95% Confidence Interval	
						Lower Bound	Upper Bound
Games-Howell	CRC	AA	1.12	2.15	.810	-3.18	5.43
		CTRL	9.42	1.99	.000	4.43	14.40
	AA	CRC	-1.12	2.15	.810	-5.43	3.18
		CTRL	8.29	2.14	.001	3.21	13.38
	CTRL	CRC	-9.42	1.99	.000	-14.40	-4.43
		AA	-8.29	2.14	.001	-13.38	-3.21

Table S2. One-way ANOVA and post-hoc analysis for gender.

Descriptives

	Group	N	Mean	Std. Deviation	Std. Error	95% Confidence Interval for Mean		Minimum	Maximum
						Lower Bound	Upper Bound		
Gende	CRC	75	.71	.46	.05	.60	.81	.00	1.00
	AA	58	.66	.48	.06	.53	.78	.00	1.00
	CTRL	78	.50	.50	.06	.39	.61	.00	1.00
	Total	211	.62	.49	.03	.55	.68	.00	1.00

Test of Homogeneity of Variances

	Levene Statistic	df1	df2	Sig.
Gende	7.45	2	208	.001

ANOVA

		Sum of Squares	df	Mean Square	F	Sig.
Gende	Between Groups	1.76	2	.88	3.79	.024
	Within Groups	48.15	208	.23		
	Total	49.91	210			

Multiple Comparisons (Gende)

	(J) Family	(J) Family	Mean Difference (I - J)	Std. Error	Sig.	95% Confidence Interval	
						Lower Bound	Upper Bound
Games-Howell	CRC	AA	.05	.08	.806	-.14	.25
		CTRL	.21	.08	.024	.02	.39
	AA	CRC	-.05	.08	.806	-.25	.14
		CTRL	.16	.08	.165	-.05	.36
	CTRL	CRC	-.21	.08	.024	-.39	-.02
		AA	-.16	.08	.165	-.36	.05

Table S3. One-way ANOVA and post-hoc analysis for fecal Hb amount in µg/g (numerical variable).

Descriptives

	Group	N	Mean	Std. Deviation	Std. Error	95% Confidence Interval for Mean		Minimum	Maximum
						Lower Bound	Upper Bound		
fob	CRC	75	685.35	421.57	48.68	588.35	782.34	19.00	1001.00
	AA	58	265.14	343.26	45.07	174.88	355.39	19.00	1001.00
	CTRL	78	104.71	246.67	27.93	49.09	160.32	19.00	1001.00
	Total	211	355.19	425.90	29.32	297.39	412.99	19.00	1001.00

Test of Homogeneity of Variances

	Levene Statistic	df1	df2	Sig.
fob	33.03	2	208	.000

ANOVA

		Sum of Squares	df	Mean Square	F	Sig.
fob	Between Groups	13539529	2	6769764	57.35	.000
	Within Groups	24553052	208	118043.5		
	Total	38092581	210			

Multiple Comparisons (FOB)

	(J) Family	(J) Family	Mean Difference (I - J)	Std. Error	Sig.	95% Confidence Interval	
						Lower Bound	Upper Bound
Games-Howell	CRC	AA	420.21	60.08	.000	262.93	577.49
		CTRL	580.64	55.56	.000	447.43	713.85
	AA	CRC	-420.21	60.08	.000	-577.49	-262.93
		CTRL	160.43	59.57	.009	34.25	286.61
	CTRL	CRC	-580.64	55.56	.000	-713.85	-447.43
		AA	-160.43	59.57	.009	-286.61	-34.25

Table S4. One-way ANOVA and post-hoc analysis for fecal Hb result (categorical variable – yes/no).

Descriptives

	Group	N	Mean	Std. Deviation	Std. Error	95% Confidence Interval for Mean		Minimum	Maximum
						Lower Bound	Upper Bound		
Var0001	CRC	75	1.13	.34	.04	1.05	1.21	1.00	2.00
	AA	58	1.33	.47	.06	1.20	1.45	1.00	2.00
	CTRL	78	1.73	.45	.05	1.63	1.83	1.00	2.00
	Total	211	1.41	.49	.03	1.34	1.47	1.00	2.00

Test of Homogeneity of Variances

	Levene Statistic	df1	df2	Sig.
Var0001	18.52	2	208	.000

ANOVA

		Sum of Squares	df	Mean Square	F	Sig.
Var0001	Between Groups	14.16	2	7.08	40.03	.000
	Within Groups	36.79	208	.18		
	Total	50.95	210			

Multiple Comparisons (FOB (yes/no))

	(J) Family	(J) Family	Mean Difference (I - J)	Std. Error	Sig.	95% Confidence Interval	
						Lower Bound	Upper Bound
Games-Howell	CRC	AA	-.19	.07	.026	-.37	-.02
		CTRL	-.60	.07	.000	-.75	-.45
	AA	CRC	.19	.07	.026	.02	.37
		CTRL	-.40	.07	.000	-.59	-.21
	CTRL	CRC	.60	.07	.000	.45	.75
		AA	.40	.07	.000	.21	.59

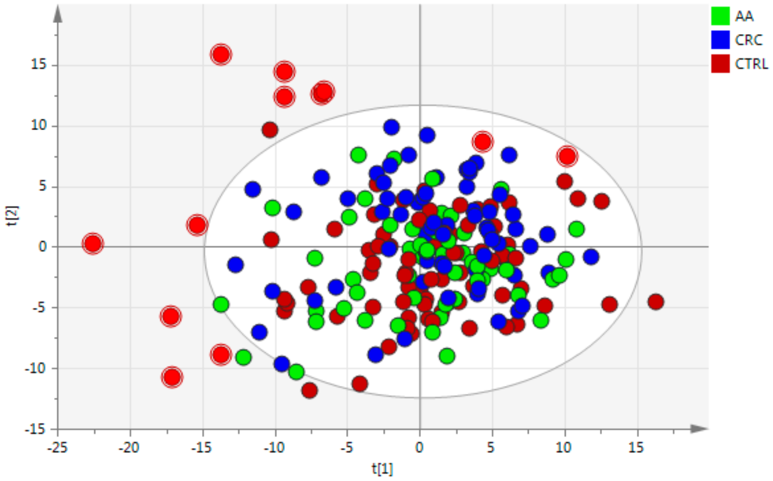

Figure S2. Scores plot of the PCA of fecal samples. Autoscaling and logarithm transformation of the data. R2X = 0.605, Q2 = 0.546, 4PCs. Highlighted red dots (samples owl-2323-001, owl-2323-016, owl-2323-019, owl-2323-034, owl-2323-067, owl-2323-068, owl-2323-071, owl-2323-105, owl-2323-109, owl-2323-127, owl-2323-148 and owl-2323-151) were outliers out of the Hotelling's T2 ellipse.

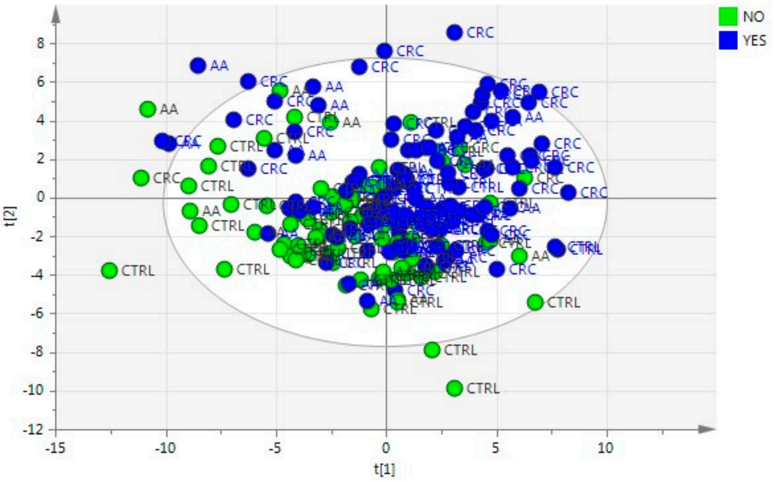

Figure S3. Scores plot of PLS-DA model of fecal samples (class label = FIT – YES (≥ 20 µg Hb/g feces) and NO (< 20 µg Hb/g feces). Autoscaling and logarithm transformation of the data. R2X = 0.465, R2Y = 0.330, Q2 = 0.155, and 3PCs. CV-ANOVA *p*-value = 4.64e-5.

Table S5. Metabolites selected from PLS-DA models with the corresponding VIP and p(corr) values when FIT (yes/no) was used as classification label.

Var ID	Metabolites	p(corr)	VIP*
ChoE_08	ChoE (18:2)	0.6501	2.0927
DG06	DG (16:0_18:1_0:0)	-0.5353	1.1797
DG07	DG (16:0_18:2_0:0)	-0.6197	1.0137
DG08	DG (18:1_18:2_0:0)	-0.6965	1.0681
DG09	DG (18:1_18:2_0:0)	-0.7320	1.0175
SphLip_10	SM (d18:1/16:0)	0.5517	1.3022
SphLip_13	SM (d18:1/18:0)	0.5327	1.1138
SphLip_20	SM (d18:1/22:0)	0.5315	1.0028
SphLip_26_SphLip_27	SM (d18:1/24:1) + SM (d18:2/24:0)	0.5904	1.2966
SphLip_30	SM (42:1)	0.5572	1.2500
TG173	TG (18:1_18:1_19:0) + TG (18:0_18:1_19:1)**	-0.5307	1.0022
TG27	TG (16:0_18:1_16:0)**	-0.6569	1.2290
TG28	TG (16:0_18:1_16:1) + TG (18:1_18:1_14:0) + TG (16:0_18:2_16:0)**	-0.6873	1.1498
TG29	TG (16:1_18:1_16:1) + TG (16:0_18:2_16:1)**	-0.7028	1.0070
TG34	TG (16:0_17:1_18:1)**	-0.6961	1.0455

TG35	TG (16:0_17:1_18:2) + TG (18:2_18:1_15:0)**	-0.6857	1.0771
TG36	TG (18:2_18:2_15:0) + TG (17:1_18:2_16:1) + TG (17:1_18:3_16:0)**	-0.6784	1.0802
TG39	TG (16:0_18:1_18:1)**	-0.6965	1.1469
TG40	TG (16:0_18:1_18:2)**	-0.7200	1.0758
TG46	TG (18:2_18:1_17:0) + TG (18:1_18:1_17:1)**	-0.6927	1.1228
TG49	TG (18:0_18:1_18:1)**	-0.7298	1.0526
TG50	TG (20:2_20:1_14:0) + TG (20:2_18:1_16:0) + TG (20:1_18:2_16:0) + TG (18:2_18:1_18:0)**	-0.7290	1.0033
TG51	TG (18:2_18:1_18:1) + TG (18:2_18:2_18:0)**	-0.7632	1.0410
TG52	TG (18:2_18:2_18:1)**	-0.7572	1.0391
TG59	TG (22:0_18:1_16:0) + TG (24:0_16:1_16:0)**	-0.6677	1.0315
TG60	TG (18:1_20:1_18:0)**	-0.7014	1.1051
TG61	TG (18:1_20:1_18:1)**	-0.7233	1.0924
TG71	TG (22:1_18:1_18:1) + TG (22:1_18:2_18:0)**	-0.6385	1.0091

* Variable Importance on Projection; ** Individual Composition or Probable ID
